# Extension of *penA* diversity within the genus *Neisseria*: characterization of a *penA/ftsI* homolog in *Neisseria bacilliformis* associated with elevated ceftriaxone MICs

**DOI:** 10.1128/aac.00392-26

**Published:** 2026-07-23

**Authors:** Sheeba Santhini Manoharan-Basil, Tessa de Block, Izumo Kanesaka, Saïd Abdellati, Thibaut Vanbaelen, Chris Kenyon

**Affiliations:** 1 Department of Clinical Sciences, Institute of Tropical Medicine Antwerp 567788 , Antwerp, Belgium; 2 Department of Infection Control and Prevention, Faculty of Nursing, Toho University, Tokyo, Japan; 3 Department of Medicine, University of Cape Town, Cape Town, South Africa; The Peter Doherty Institute for Infection and Immunity, Melbourne, Victoria, Australia

**Keywords:** penA, ftsI, *Neisseria bacilliformis*, *Neisseria gonorrhoeae*, ceftriaxone

## Abstract

Reduced susceptibility to extended-spectrum cephalosporins in *Neisseria gonorrhoeae* is primarily mediated by structural alterations in penicillin-binding protein 2 (PBP2), encoded by *penA*, frequently arising through interspecies recombination with commensal *Neisseria*. While mosaic *penA* diversity has been extensively characterized in coccoid *Neisseria* species, rod-shaped commensals such as *Neisseria bacilliformis* remain poorly explored. Here, we investigated the β-lactam target repertoire of *N. bacilliformis* using whole-genome sequencing, comparative genomics, mutation profiling, and structural modeling. Fifteen *N. bacilliformis* genomes were analyzed, including eight isolates with elevated ceftriaxone minimum inhibitory concentrations (MIC) (range 0.094–1 µg/mL; median 0.5 µg/mL). Interrogation of the PubMLST locus NEIS1753 did not identify matches under default parameters. However, *de novo* annotation revealed a syntenic high-molecular-mass transpeptidase annotated as *ftsI*, occupying the conserved *rsmH-ftsL-ftsI-murE* chromosomal context observed in *N. gonorrhoeae*. BLASTP analysis demonstrated ~59%–61% amino-acid identity to gonococcal PBP2. Structural modeling using AlphaFold and template-based approaches confirmed preservation of the canonical class B penicillin-binding protein fold, including conserved catalytic motifs. Across 24 positions previously associated with altered cephalosporin susceptibility in gonococci, multiple canonical substitutions were conserved in *N. bacilliformis*, alongside lineage-specific variants not reported in *N. gonorrhoeae*. Interestingly, polymorphism at positions A328 and A341 was observed in isolates with higher MICs (1 µg/mL). Definitive genotype-phenotype associations could not be established. Recombination analysis did not identify discrete parental donors but suggested a complex evolutionary history. These findings demonstrate that *N. bacilliformis* encodes a structurally conserved PBP2 homolog annotated as FtsI and expand the landscape of β-lactam target diversity within the genus *Neisseria*.

## INTRODUCTION

The genus *Neisseria* comprises a diverse group of gram-negative, oxidase- and catalase-positive bacteria that colonize epithelial surfaces of humans and animals (1, 2). Among the human-restricted species, two principal morphologies are observed: coccoid (*Neisseria cinerea*, *Neisseria gonorrhoeae*, *Neisseria polysaccharea*, *Neisseria lactamica*, *Neisseria meningitidis*, *Neisseria mucosa*, *Neisseria oralis*, and *Neisseria subflava*) and rod-shaped organisms (*Neisseria elongata* and *Neisseria bacilliformis*) (3–9). Most members of the genus are commensals of the oro- and nasopharyngeal mucosa. However, *N. meningitidis* and *N. gonorrhoeae* are pathogens capable of causing a range of infections, including urethritis, cervicitis, meningitis, septicemia, and disseminated gonococcal disease (10–12).

The genome of *N. gonorrhoeae* encodes five penicillin-binding proteins (PBPs), comprising two essential high-molecular-mass (HMM) transpeptidases and three low-molecular-mass (LMM) class C enzymes (13). The HMM PBPs include PBP1 (*ponA*), homolog of *Escherichia coli* PBP1a (*mrcA*), a class A bifunctional enzyme responsible for both *trans* glycosylation and transpeptidation during cell growth and elongation, and PBP2 (*penA*), homolog of *E. coli* PBP3 (*ftsI*), a class B septal transpeptidase essential for peptidoglycan cross-linking during cell division (14, 15). In contrast, the three LMM PBPs—PBP3 (*dacB*), PBP4 (*pbpG*), and DacC—primarily exhibit carboxypeptidase and/or endopeptidase activity and are not essential for viability but contribute to cell wall remodeling and morphology (16, 17).

PBP2, encoded by *penA*, is the principal determinant of extended-spectrum cephalosporin susceptibility and constitutes the primary β-lactam target in *N. gonorrhoeae* (18, 19). Ceftriaxone remains the first-line therapy for *N. gonorrhoeae*; however, its clinical efficacy is increasingly threatened by the global emergence and dissemination of strains with reduced susceptibility (20). Decreased susceptibility is predominantly mediated by structural alterations in PBP2, most notably through the evolution of mosaic *penA* alleles generated via interspecies recombination with commensal *Neisseria* species (21–24). Within genomic surveillance frameworks, *penA* is curated in the PubMLST *Neisseria* database as locus NEIS1753, and recent population-level analyses have comprehensively mapped mosaic *penA* diversity and its commensal origins across multiple *Neisseria* species within the genus (25). Nevertheless, rod-shaped commensals, including *N. elongata* and *N. bacilliformis*, remain underrepresented in this analysis.

Previous studies have substantially advanced our understanding of how commensal *Neisseria* contributes to the evolutionary reservoir of *penA* diversity. Building on these analyses, exploration of *penA* homologs in additional *Neisseria* species may offer complementary insights, particularly where functionally equivalent loci are annotated under alternative gene names across the genus. *N. bacilliformis* is an oropharyngeal commensal species that has not been previously included in systematic analyses of *penA* diversity (25). As clinical breakpoints for ceftriaxone susceptibility have not been established for *N. bacilliformis*, EUCAST interpretive criteria defined for *N. gonorrhoeae* were applied when evaluating minimum inhibitory concentrations (MICs). Recent commensal surveillance in Belgium reported comparatively high ceftriaxone MICs in *N. bacilliformis* (median 0.5 mg/L; IQR 0.38–0.75) (26). In addition, a clinical *N. bacilliformis* isolate (isolate id: AR-0943) also showed high ceftriaxone resistance (MIC 16 mg/L) (26, 27). Despite these observations, the contribution of *N. bacilliformis* to the diversity and evolution of penicillin-binding proteins within the genus remains unexplored. Whether this species harbors a *penA* equivalent locus, and how such a gene relates structurally and evolutionarily to canonical gonococcal PBP2, has not previously been investigated.

In this study, we use long-read whole-genome sequencing, comparative genomics, and structural modeling to characterize a *penA*/*ftsI* homolog in *N. bacilliformis*. We show that this species encodes a high-molecular-mass transpeptidase annotated as *ftsI* by Prokka annotation tool (28). The gene occupies a chromosomal context syntenic to gonococcal *penA* and exhibits strong structural homology to PBP2. Furthermore, recombination detection analysis did not identify statistically supported parental donors, although sequence similarity patterns suggest a complex evolutionary history that cannot be fully resolved with currently available genomes. Notably, the *N. bacilliformis* transpeptidase shares several amino-acid substitutions with gonococcal PBP2 at positions previously associated with decreased cephalosporin susceptibility. Together, these findings extend current frameworks describing penicillin-binding protein diversity within the genus *Neisseria*.

## MATERIALS AND METHODS

### Study isolates and whole-genome sequencing

Eight *N. bacilliformis* isolates previously identified during commensal *Neisseria* surveillance in Belgium with high ceftriaxone (CRO) MIC (Table S1) were selected for whole-genome sequencing (26).

Genomic DNA was extracted from a single colony using the MasterPure Complete DNA and RNA Purification Kit (Epicenter, Madison, USA), according to the manufacturer’s instructions. Sequencing libraries were prepared with the Oxford Nanopore Technologies Native Barcoding Kit (SQK-NBD114.24) and sequenced on a MinION R10.4 flow cell. The R10 chemistry sequence accuracy is comparable to Illumina platforms and enables stable phylogenetic reconstruction, making it suitable for detecting single-nucleotide polymorphism (29). Base calling was performed with Dorado (v0.9.1; https://github.com/nanoporetech/dorado) using the super-accuracy (sup) model. *De novo* genome assemblies were generated using Autocycler (v0.3.0) (30) and polished using Dorado polish with --bacteria parameter. Assembly quality was assessed using QUAST (v5.3.0) (31). Final assemblies were uploaded to the PubMLST Neisseria database (https://pubmlst.org/organisms/neisseria-spp) for locus interrogation and allele assignment. The IDs of the assemblies in PubMLST are as follows: 169223, 169224, 169225, 169226, 169227, 169228, 169229, and 169230.

### Gene annotation and locus interrogation

*De novo* annotation was performed using Prokka (v1.14.6) with default parameters (28). To identify homologs of the canonical gonococcal *penA* locus, the PubMLST *Neisseria* database (https://pubmlst.org/organisms/neisseria-spp) was used, where *penA* is curated as locus NEIS1753. Assembled *N. bacilliformis* genomes were uploaded to PubMLST and initially queried using the default parameters, which resulted in “no-hits” to NEIS1753. A secondary search strategy was then applied using less stringent similarity thresholds (Min %identity: 30, Min % alignment: 10, and BLASTn wordsize: 7) to recover divergent homologs. Candidate loci identified through this approach were further examined through manual inspection and comparative annotation.

### Structural modeling and comparative analysis

The full-length FtsI amino-acid sequence from *N. bacilliformis* was subjected to structural prediction using AlphaFold with default parameters (32). Model confidence was assessed using predicted TM-score (pTM), per-residue predicted local distance difference test (pLDDT) values, and predicted aligned error (PAE) matrices. Independent template-based modeling was performed using SWISS-MODEL (33). Template selection was based on sequence identity, coverage, and model quality metrics, including Global Model Quality Estimation (GMQE) and QMEANDisCo scores.

To assess chromosomal context, the genomic region spanning *rsmH-ftsL*-[*penA/ftsI*]-*murE* was extracted from each assembly and compared with *N. gonorrhoeae* FA1090. Gene order and orientation were inspected using CLC Genomics Workbench v25.

### Dataset used, allele identification, and representative sequence selection for recombination analysis

In the current study, eight *N. bacilliformis* were sequenced, from which the *ftsI*-annotated *penA* homolog was extracted and combined with seven publicly available *N. bacilliformis* genomes retrieved from NCBI, resulting in a total of fifteen WGS for *N. bacilliformis*. For *N. gonorrhoeae*, all *penA* alleles (*n* = 899) were downloaded from NG-STAR database (https://ngstar.canada.ca/alleles/loci_selection?lang=en). *N. meningitidis* sequences (*n* = 46,446) corresponding to locus NEIS1753 were exported from the PubMLST database (https://pubmlst.org/organisms/neisseria-spp). In addition, *penA* sequences from commensal *Neisseria* species (*n* = 115) were obtained from a previously published commensal *Neisseria* surveillance study in Belgium (34).

To reduce redundancy and computational burden for recombination detection, *penA* nucleotide sequences from *N. meningitidis* data set were clustered using cd-hit-est v4.8.1 at 99% nucleotide identity with a word size of 10 (-c 0.99 -n 10) (35). For each cluster, the centroid sequence selected by cd-hit-est was retained as the representative sequence, while all other sequences sharing ≥99% identity were designated as cluster members. Representative identifiers were extracted from the cd-hit-est output and used to retrieve the corresponding aligned sequences from the multiple sequence alignment generated using MAFFT (v7.453) (36). This resulted in a reduced, non-redundant aligned data set of 611 *N. meningitidis* sequences suitable for recombination analysis. Cluster information was parsed from the cd-hit-est.clstr file and summarized in a mapping table linking each sequence to its cluster and representative status.

The final data set (*n* = 1,640) used for recombination detection composed of 15 *N. bacilliformis* sequences, 899 *N. gonorrhoeae* alleles, 115 commensal *Neisseria* sequences, and 611 non-redundant *N. meningitidis* sequences. The recombination analysis was carried out as described in reference 37. In brief, the Recombination Detection Program (RDP5; v.5.7.5) was used to carry out the analysis (38, 39). Recombinant events supported by at least two of the nine algorithms available in RDP software: RDP, GENECONV, Bootscan, Maxchi, Chimera, SiSscan, PhyloPro, LARD, and 3Seq were used with default settings (38, 40). According to the definition in RDP5, the minor parent is the parental sequence that contributes the smaller fraction of the recombinant sequence, while the major parent is the parental sequence that contributes the larger fraction (38)

### Detection of amino-acid substitutions

Amino-acid substitutions within the *penA/ftsI* alignment were identified through comparison of translated sequences against the reference *N. gonorrhoeae* FA1090 PBP2 sequence. Alignment positions were mapped to FA1090 amino-acid coordinates, CARD ARO:3004832 reference numbering, and corresponding *N. bacilliformis* positions to ensure consistent cross-species positional comparison. The data set for this analysis is composed of 15 *N. bacilliformis* and 899 *N. gonorrhoeae penA* sequences.

Substitutions previously associated with reduced cephalosporin susceptibility were compiled from three independent sources: (i) Zhang et al (41), (ii) van Hal et al (42), and (iii) the Comprehensive Antibiotic Resistance Database (CARD) (43). Each reported position was manually cross-referenced against the multiple sequence alignment to confirm positional equivalence across species. Presence or absence of the corresponding amino-acid substitution in *N. bacilliformis* and comparator *Neisseria* data sets was manually recorded. Positions were categorized according to mosaic or non-mosaic association based on established gonococcal *penA* classifications. For each site, amino-acid substitution was annotated using FA1090 numbering (e.g., V279A, E285D, K288R, Q291R, A311V, I312M, V316P, A328P, A341P, T483S, T485I, A459T, A501P/T/V, F504LV, A510V, N512Y, A516G, H541N, G542S, G545S, P551L/S, P552V, K555Q, and I556V). Both alignment coordinates and species-specific amino-acid coordinates were retained to enable transparent cross-referencing between data sets. This approach ensured harmonized positional mapping across studies and allowed systematic interrogation of amino-acid substitutions reported in the literature within the context of the *N. bacilliformis* locus. In the present study, amino-acid substitutions previously reported in association with altered cephalosporin susceptibility and documented in the literature or curated resistance databases were classified as canonical substitutions, and amino-acid substitutions identified that were not previously reported in these sources were designated as non-canonical substitutions.

**Fig 1:**
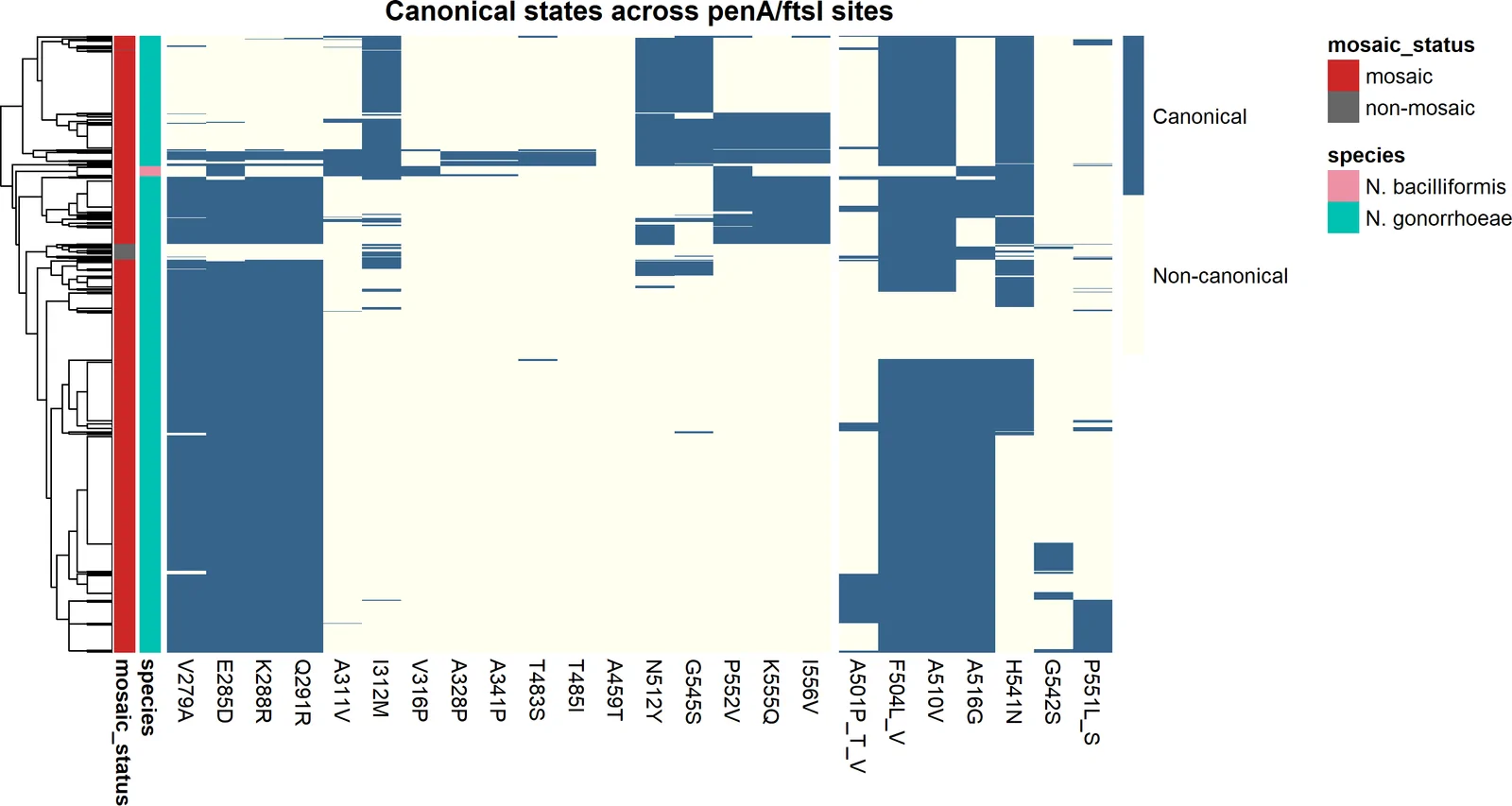
Amino-acid substitution status across penA/ftsI sites in *Neisseria bacilliformis* and *Neisseria gonorrhoeae*. Heatmap showing amino-acid substitution status at 24 penA/ftsI positions previously associated with altered cephalosporin susceptibility in gonococci. Rows represent 15 *N. bacilliformis penA/ftsI* sequences and 899 *N. gonorrhoeae PenA* sequences. Blue indicates canonical substitutions previously reported in gonococcal *penA* alleles, whereas ivory indicates residues not classified as canonical at that position, including wild-type or alternative non-canonical residues. These categories describe amino-acid identity only and do not indicate phenotypic resistance status.

**Fig 2:**
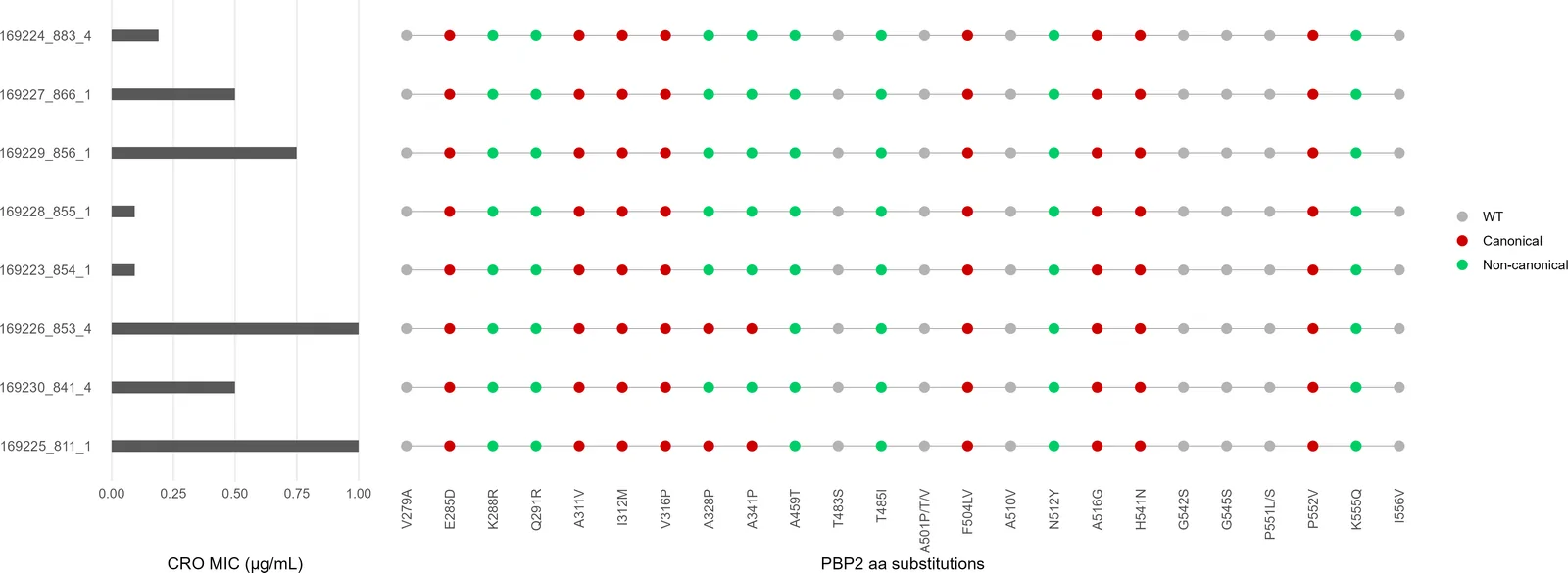
Distribution of PBP2/FTSI amino-acid substitutions across *N. bacilliformis* isolates with corresponding ceftriaxone MICs. Each row represents a single isolate (*n* = 8), and each column represents one of the 24 interrogated amino-acid positions within PBP2, labeled by wild-type amino acid, position, canonical, and non-canonical substitutions. Dots indicate the observed amino acid at each position. Gray dots represent wild-type amino acids, red dots represent canonical substitutions previously associated with altered cephalosporin susceptibility, and green dots represent non-canonical substitutions not previously reported. The left panel shows the corresponding ceftriaxone minimum inhibitory concentration (MIC, µg/mL) for each isolate.

Figure 1 and 2 were generated using RStudio (2025.05.1) using custom scripts.

## RESULTS

### Genome sequencing and identification of a *penA/ftsI* homolog in *N. bacilliformis*

Long-read whole-genome sequencing was performed for eight *N. bacilliformis* isolates. *De novo* annotation using Prokka identified a high-molecular-mass class B transpeptidase annotated as *ftsI* in all isolates. No locus was annotated as *penA*. Interrogation of the PubMLST *Neisseria* database, where gonococcal *penA* is curated as locus NEIS1753, did not yield matches under default allele-matching parameters when *N. bacilliformis* assemblies were uploaded and queried. However, relaxed similarity thresholds recovered a divergent homolog corresponding to the Prokka-annotated *ftsI* locus.

### Structural conservation of the FtsI transpeptidase domain

AlphaFold modeling predicted a well-defined global fold for *N. bacilliformis* FtsI (pTM = 0.82), indicating high confidence in overall domain architecture. The core transpeptidase domain exhibited uniformly high per-residue confidence (pLDDT > 90), whereas reduced confidence was confined to the distal C-terminal region, consistent with flexible or membrane-associated segments typical of FtsI homologs (Fig. S1). The PAE matrix demonstrated strong local structural reliability across the catalytic domain.

Template-based modeling using SWISS-MODEL independently corroborated these findings. Among 1,333 candidate templates, the top-ranked structure corresponded to a peptidoglycan D,D-transpeptidase FtsI homolog (92.19% sequence identity; GMQE = 0.88), indicating high predicted structural accuracy. Crystal structures of *N. gonorrhoeae* PBP2 (PDB: 5KSH, 3EQV) were also identified among the highest-ranking templates, sharing approximately 60%–61% sequence identity across the aligned region.

### Conservation of chromosomal context and synteny

Comparative analysis of chromosomal synteny demonstrated that the *N. bacilliformis ftsI* locus is positioned within a conserved genomic region spanning the gene order *rsmH-ftsL-[ftsI/penA]-murE*. This mirrors the gene order observed in *N. gonorrhoeae* FA1090 (*rsmH-ftsL-penA-murE*), supporting positional orthology between *ftsI* in *N. bacilliformis* and gonococcal *penA*. No large-scale rearrangements or inversions were observed within this locus across the 15 genomes analyzed.

### Amino-acid substitutions at positions associated with altered cephalosporin susceptibility

Canonical and non-canonical amino acid substitutions across *penA*/ftsI sites in *N. bacilliformis* and *N. gonorrhoeae* are shown in Fig. 1, with corresponding sequence alignment provided in Material S1.

A total of 24 amino-acid substitution sites previously reported in association with altered cephalosporin susceptibility were interrogated across the 15 *N. bacilliformis* genomes. Among mosaic-associated positions, several canonical substitutions were uniformly present in all isolates (15/15), including E285D, A311V, I312M, V316P, A459T, and P552V (Table 1). Additional canonical substitutions classified as non-mosaic in gonococci (F504V and A516G) were also conserved across all isolates. In contrast, a subset of substitutions is not currently reported in gonococcal *penA* alleles, including K288E, Q291E, T485R, N512D, and K555N (Table 1). These substitutions were conserved across all 15 *N. bacilliformis* isolates.

**TABLE 1:** Distribution of amino-acid substitutions in the *N. bacilliformis* FtsI transpeptidase at 24 positions previously associated with altered cephalosporin susceptibility in gonococcal PBP2^*a*^

Known aa substitutions	penA mosaicism	Observed aa substitutions in *N. bacilliformis*	Number of *N. bacilliformis* isolates
V279A	Mosaic	V	15
E285D	Mosaic	D	15
K288R	Mosaic	E	15
Q291R	Mosaic	E	15
A311V	Mosaic	V	15
I312M	Mosaic	M	15
V316P	Mosaic	P	15
A328P	Mosaic	P	3
		T	12
A341P	Mosaic	P	3
		T	12
T483S	Mosaic	T	15
T485I	Mosaic	R	15
A459T	Mosaic	E	15
A501P/T/V	Non-mosaic	A	15
F504LV	Non-mosaic	V	15
A510V	Non-mosaic	A	15
N512Y	Mosaic	D	15
A516G	Non-mosaic	G	15
H541N	Non-mosaic	N	15
G542S	Non-mosaic	G	15
G545S	Mosaic	G	15
P551L/S	Non-mosaic	P	15
P552V	Mosaic	V	15
K555Q	Mosaic	N	15
I556V	Mosaic	I	15

^*a*^ Substitutions were classified as canonical when previously reported in *Neisseria gonorrhoeae* penA alleles and as non-canonical when not documented in gonococcal datasets. Sites identical to the *N. gonorrhoeae* FA1090 reference residue were designated wild type (WT). The number of *N. bacilliformis* isolates (*n* = 15) harboring each substitution is indicated.

At positions A328 and A341, intra-species polymorphism was observed. The canonical substitutions A328P and A341P were detected in the same 3/15 isolates, whereas 12/15 isolates encoded threonine at each respective position (Table 1). The A328P variant was observed in 21/899 gonococcal alleles (Table S2). Ceftriaxone MIC data were available only for the eight newly sequenced *N. bacilliformis* isolates; therefore, Fig. 2 was restricted to these isolates to allow direct visualization of amino acid substitution profiles alongside isolate-specific MIC values. Of the three *N. bacilliformis* isolates harboring the A328P/A341P substitutions, two originated from the Belgian *Neisseria* commensal surveillance study and had ceftriaxone MICs of 1 mg/L (Fig. 2 and Tables S1 and S2). Several positions retained the FA1090 reference residue (WT) across all isolates, including V279, T483, A501, A510, G542, G545, P551, and I556.

### Recombination analysis

Recombination detection using RDP5 across 1,640 aligned *penA/ftsI* nucleotide sequences identified 544 putative recombination events. Five of these events (342 and 541–544) involved *N. bacilliformis* sequences (Table S3). However, none were supported by statistically significant *P*-values across the nine detection algorithms. For example, event 542 had no significant *P*-value, and *N. meningitidis* (71530|NZ16MI0023|New_Zealand|2016) was the recombinant and had corresponding minor parent *N. bacilliformis* (169223_854_1) and major parent *N. gonorrhoeae* (penA_344.001; Table S3). Several breakpoint positions were marked as undetermined or potentially confounded by overlapping events. However, in several cases, inferred parental assignments pointed to unknown parents, suggesting that broader genomic sampling of rod-shaped and related commensal *Neisseria* species may be required to fully resolve the evolutionary origins.

## DISCUSSION

In this study, we identify a high-molecular-mass class B transpeptidase in *Neisseria bacilliformis* annotated as *ftsI* that is syntenic with gonococcal *penA* and structurally homologous to PBP2. Although no allele matches were detected under default PubMLST interrogation of locus NEIS1753, relaxed similarity searches, comparative genomic, and structural analyses establish this locus as the PBP2 ortholog in *N. bacilliformis*, embedded within the conserved *rsmH-ftsL-ftsI-murE* genomic context. This annotation is consistent with the established homology between gonococcal PBP2, encoded by *penA*, and *Escherichia coli* PBP3, encoded by *ftsI*, and therefore reflects annotation nomenclature rather than the absence of a *penA*-equivalent locus (14, 15). Thus, the absence of a default NEIS1753 match should be interpreted as an annotation and database-match limitation rather than the absence of a *penA*-equivalent β-lactam target. The high conservation observed across the *N. bacilliformis ftsI/penA* homolog further supports its classification as an essential class B transpeptidase ortholog. Together, these findings extend current frameworks describing β-lactam target evolution across the genus *Neisseria* and underscore the importance of annotation-aware comparative genomics.

Alterations in PBP2 are the principal determinant of reduced susceptibility to extended-spectrum cephalosporins in *N. gonorrhoeae*, particularly through mosaic *penA* alleles generated by interspecies recombination (15, 25, 44–46). While large-scale genomic analyses have delineated the commensal origins of mosaic *penA* diversity in coccoid *Neisseria* (25), rod-shaped taxa such as *N. bacilliformis* have remained comparatively unexplored. Our analysis demonstrates that *N. bacilliformis* harbors multiple amino acid substitutions at positions previously implicated in altered cephalosporin susceptibility, including canonical variants described in gonococcal *penA* alleles as well as lineage-specific substitutions not observed in *N. gonorrhoeae*. These findings expand the evolutionary landscape of class B transpeptidase diversity within the genus *Neisseria*.

Structural modeling using AlphaFold and template-based approaches confirmed preservation of the canonical class B PBP architecture, including conserved catalytic motifs and active-site configuration. The high-confidence transpeptidase core supports the interpretation that substitutions identified in *N. bacilliformis* occur within a structurally conserved scaffold analogous to gonococcal PBP2. This conservation aligns with the essential role of FtsI/PBP2 in septal peptidoglycan cross-linking during cell division (14, 15).

Recent evolutionary analyses of Neisseriaceae further contextualize these findings. Rod-shaped members, including species closely related to *N. bacilliformis*, undergo longitudinal rather than transverse division while retaining core divisome components such as FtsZ and FtsI (47). Morphological innovation within the family therefore appears to arise through spatial and regulatory reorganization of the divisome rather than replacement of essential peptidoglycan synthesis enzymes. The structural conservation of FtsI observed here supports the notion that β-lactam targets remain evolutionarily conserved even in morphologically divergent lineages.

Across 24 positions previously associated with altered cephalosporin susceptibility in gonococci, *N. bacilliformis* exhibited both canonical and non-canonical substitutions. Several canonical substitutions frequently observed in mosaic gonococcal *penA* alleles, including A311V, I312M, V316P, A459T, F504V, A516G, and P552V, were uniformly conserved across isolates. However, these substitutions were not organized into clustered mosaic segments characteristic of highly resistant gonococcal alleles (25, 46, 48), suggesting that their structural context differs in *N. bacilliformis*. Consistent with functional studies in gonococci demonstrating that elevated ceftriaxone MICs arise from cumulative structural perturbations and epistatic interactions rather than isolated substitutions (14, 19), the presence of individual canonical variants in *N. bacilliformis* does not imply equivalent phenotypic consequences.

Emerging reports of elevated ceftriaxone MICs in *N. bacilliformis* underscore the relevance of characterizing β-lactam targets in this species (26). At positions A328 and A341, intra-species polymorphism was observed. The canonical A328P and A341P substitutions were present in 3/15 isolates, while the remaining isolates encoded threonine at both positions. The A328P variant has been reported in 21 of 899 gonococcal *penA* alleles, indicating that although uncommon, it is not unique to *N. bacilliformis*. Notably, two *N. bacilliformis* isolates harboring the A328P/A341P configuration exhibited the highest ceftriaxone MICs in our data set (1 mg/L). These residues localize within the transpeptidase domain, raising the possibility that local conformational effects may influence substrate or β-lactam interactions. However, given the limited sample size and shared substitution background, a direct genotype-phenotype relationship cannot be inferred. Targeted mutagenesis and β-lactam binding assays will be necessary to determine functional impact.

Several substitutions identified in *N. bacilliformis*, including K288E, Q291E, T485R, N512D, and K555N, have not been reported in gonococcal *penA* alleles and likely represent lineage-specific diversification of the class B transpeptidase scaffold. Their evolutionary significance remains unclear but may reflect adaptive processes distinct from those driving mosaic *penA* evolution in *N. gonorrhoeae*.

Although numerous recombination events were detected across the broader *Neisseria* data set, only a small subset (*n* = 5 events) involved *N. bacilliformis*, and none were statistically significant across the nine methods. Nevertheless, mosaic *penA* alleles in *N. gonorrhoeae* are known to arise via interspecies recombination with commensal *Neisseria* species (25, 44). The absence of discrete breakpoints in *N. bacilliformis* suggests limited sampling of rod-shaped taxa, and thus, expanded sampling of rod-shaped commensals may be required to resolve the evolutionary exchange.

Notably, isolation of *N. bacilliformis* in the study by Abdellati et al. was facilitated by the use of oral rinse sampling, consistent with previous observations that this approach increases recovery of commensal *Neisseria* compared to conventional swab-based methods (26). Incorporation of oral rinse sampling into future surveillance frameworks may therefore improve detection of morphologically and ecologically distinct commensal species that could contribute to the broader β-lactam resistance reservoir.

A further limitation of the study is that the amino-acid substitution analysis in the present study is relative to *N. gonorrhoeae*, because the canonical substitutions interrogated here were derived from gonococcal ceftriaxone-resistance literature. Although *N. meningitidis* NEIS1753 sequences and commensal *Neisseria* sequences were included in the recombination data set, broader genus-wide comparison of substitution frequencies across all *Neisseria* species was beyond the scope of this study. Such analyses will be important in future work to determine whether substitutions classified here as non-canonical relative to *N. gonorrhoeae* occur in other commensal or pathogenic *Neisseria* lineages.

Our findings broaden the evolutionary landscape of β-lactam target diversity within the genus *Neisseria* and underscore the importance of annotation-aware comparative genomics for identifying homologous resistance-associated loci across species. However, the limited number of currently available *N. bacilliformis* genomes constrains population-level evolutionary inference. Expanded genomic surveillance of rod-shaped commensal species will therefore be essential to define their contribution to the broader reservoir of β-lactam target diversity with potential relevance to antimicrobial resistance in pathogenic *Neisseria*.

## Data Availability

The raw reads are available under NCBI BioProject PRJNA1471610.

## SUPPLEMENTAL MATERIAL

Data S1 aac.00392-26-s0001.fasta Multiple sequence alignment of the FtsI/PBP2 homolog region in *Neisseria bacilliformis* and *Neisseria gonorrhoeae* penA alleles.

Fig. S1 aac.00392-26-s0002.tif AlphaFold-predicted tertiary structure of the *Neisseria bacilliformis* FtsI transpeptidase, colored by pLDDT.

Supplemental material aac.00392-26-s0003.docx Supplemental figure and table legends.

Table S1 aac.00392-26-s0004.xlsx *Neisseria bacilliformis* isolates included in this study with corresponding ceftriaxone MIC values.

Table S2 aac.00392-26-s0005.xlsx Comprehensive amino-acid substitutions for the *N. bacilliformis* FtsI protein relative to the *N. gonorrhoeae* FA1090 PBP2 reference sequence.

Table S3 aac.00392-26-s0006.xlsx Summary of recombination events detected by RDP5 analysis.

ASM does not own the copyrights to Supplemental Material that may be linked to, or accessed through, an article. The authors have granted ASM a non-exclusive, world-wide license to publish the Supplemental Material files. Please contact the corresponding author directly for reuse.
