# Supplementary material for: Extension of penA diversity within the genus Neisseria: characterization of a penA/ftsI homolog in Neisseria bacilliformis associated with elevated ceftriaxone MICs
Source: Antimicrob Agents Chemother. 2026 Jul 23;70(9):e00392-26. doi: 10.1128/aac.00392-26 (PMC13713004; doi:10.1128/aac.00392-26)
Supplement: Supplementary file 1 [file aac.00392-26-s0003.docx]

**Supplementary Table 1:** *Neisseria bacilliformis* isolates included in this study with corresponding ceftriaxone minimum inhibitory concentration (MIC) values. MICs were determined using E-test methodology and are reported in µg/mL. Clinical breakpoints are not established for *N. bacilliformis*, interpretation was performed using EUCAST breakpoints defined for *Neisseria gonorrhoeae*.

**Supplementary Table 2:** Comprehensive amino-acid substitutions for the *N. bacilliformis* FtsI protein relative to the *N. gonorrhoeae* FA1090 PBP2 reference sequence. Alignment positions were harmonized to FA1090 numbering. Positions previously associated with mosaic or non-mosaic gonococcal *penA* alleles along with the references are indicated.

**Supplementary Table 3:** Summary of recombination events detected by RDP5 analysis. The table shows recombination events identified among 1,640 aligned sequences, including events involving *N. bacilliformis*. Statistical support across detection algorithms is indicated.

**Supplementary Figure 1**: AlphaFold-predicted tertiary structure of the *Neisseria bacilliformis* FtsI transpeptidase, colored by per-residue confidence (pLDDT). Dark blue indicates very high confidence (pLDDT > 90), cyan confident regions (70-90), yellow low confidence (50-70), and orange very low confidence (<50). The core transpeptidase domain exhibits uniformly high confidence, consistent with preservation of the canonical class B penicillin-binding protein architecture. Lower-confidence regions are confined primarily to terminal segments, consistent with flexible or membrane-associated regions characteristic of *FtsI* homologs. The predicted global model confidence (pTM = 0.82) supports reliable overall domain organization.

**Supplementary material 1:** Multiple sequence alignment of the FtsI/PBP2 homolog region in *Neisseria bacilliformis* and *Neisseria gonorrhoeae* *penA* alleles. Amino-acid alignment is generated in CLC Genomics Workbench.
